# The protective role of CD73 in periodontitis: preventing hyper-inflammatory fibroblasts and driving osteoclast energy metabolism

**DOI:** 10.3389/froh.2023.1308657

**Published:** 2023-12-13

**Authors:** Erivan S. Ramos-Junior, Shantiece Dawson, Weston Ryan, Braden Clinebell, Rogelio Serrano-Lopez, Marsha Russell, Rylee Brumbaugh, Roger Zhong, Jussara Gonçalves Fernandes, Luciana M. Shaddox, Christopher W. Cutler, Ana Carolina Morandini

**Affiliations:** ^1^ Department of Oral Biology and Diagnostic Sciences, Dental College of Georgia, Augusta University, Augusta, GA, United States; ^2^ Department of Neuroscience and Regenerative Medicine, Medical College of Georgia, Augusta University, Augusta, GA, United States; ^3^ Division of Periodontology and Center for Oral Health Research, College of Dentistry, University of Kentucky, Lexington, KY, United States; ^4^ Department of Periodontics, Dental College of Georgia, Augusta University, Augusta, GA, United States

**Keywords:** periodontitis, inflammation, osteoclast, fibroblast, metabolism, CD73, mitochondrion

## Abstract

**Introduction:**

Periodontitis is an immune-mediated inflammatory disease affecting almost half of the adult population and is the leading cause of tooth loss in the United States. The role of extracellular nucleotide signaling including nucleotide metabolizing enzyme CD73 adds an important layer of interaction of purine mediators capable of orchestrating inflammatory outcomes. CD73 is able to catabolize 5′-adenosine monophosphate into adenosine at the extracellular level, playing a critical role in regulating many processes under physiological and pathological conditions. Here, we explored the role of CD73 in ligature-induced periodontitis *in vivo* comparing wild-type C57Bl/6J and CD73-deficient mice.

**Methods:**

We assessed gingival levels of inflammatory cytokines *in vivo* and in murine gingival fibroblasts *in vitro*, as well as bone loss, and RANKL-induced osteoclastogenesis. We have also analyzed CD73 mRNA in samples derived from patients diagnosed with severe periodontitis.

**Results:**

Our results in mice show that lack of CD73 resulted in increased inflammatory cytokines and chemokines such as IL-1β, IL-17, Cxcl1 and Cxcl2 in diseased gingiva relative to the healthy-controls and in comparison with the wild type. CD73-deficient gingival fibroblasts also manifested a defective healing response with higher MMP-13 levels. CD73-deficient animals also showed increased osteoclastogenesis *in vitro* with increased mitochondrial metabolism typified by excessive activation of oxidative phosphorylation, increased mitochondrial membrane potential and accumulation of hydrogen peroxide. Micro-CT analysis revealed that lack of CD73 resulted in decreased bone mineral density, decreased trabecular bone volume and thickness as well as decreased bone volume in long bones. CD73 deficiency also resulted in increased alveolar bone loss in experimental periodontitis. Correlative studies of gingival samples from severe (Grade C) periodontitis showed decreased levels of CD73 compared to healthy controls, further supporting the relevance of our murine results.

**Conclusion:**

In conclusion, CD73 appears to play a protective role in the gingival periodontal tissue and bone homeostasis, regulating hyper-inflammatory state of stromal fibroblasts and osteoclast energy metabolism and being an important candidate for future target therapies to prevent or control immune-mediated inflammatory and osteolytic diseases.

## Introduction

Although periodontitis is a chronic inflammatory disease, presenting a late onset in the majority of the patients, our knowledge about the mediators and markers associated with more severe manifestation of the disease and hyper-inflammatory responses leading to osteolytic destruction are unclear. In this context, the influence of nucleotide metabolizing enzymes such as ecto-5′-nucleotidase (NT5E), also known as CD73, characterizing purinergic influence on inflammatory alveolar bone loss is very limited. CD73 is a cell surface marker that generates adenosine, which can be critical to resolve persistent inflammation by binding to adenosine receptors. It has been demonstrated CD73-generated extracellular adenosine promotes resolution of neutrophil-mediated tissue injury (1), being generally regarded as a homeostatic mediator against tissue damage and stress conditions (2).

Regarding bone biology, CD73/adenosine signaling is a key mechanism of osteoblast differentiation and activation during development and murine bone homeostasis (3). Previous study in CD73 ^−/−^ knockout mouse model, revealed an essential role for CD73 in bone healing of tibial defect in aged mice (4). The metabolic profile of bone resorptive cells is a field of intensive investigation in osteoclast biology (5). Because of high energy demand of bone-resorbing activity, osteoclasts rely on mitochondrial metabolism as the main source of reactive oxygen species which are used to fuel RANKL-induced differentiation and activation (6).

In this study, we discovered gingival fibroblast hyper-inflammatory response and osteoclast mitochondrial metabolism and activity is highly dependent on CD73, ultimately affecting the magnitude of bone loss. Our approach consisted of cause-and-effect studies in CD73 knockout *in vivo* model and loss of function *in vitro* analysis, combined with human correlative studies using severe periodontitis and healthy samples. Our results suggest that CD73 plays a protective role in bone metabolism and by controlling inflammation-associated gingival fibroblast state. We also demonstrated the importance of CD73 in mitochondrial metabolic function driving RANKL-induced osteoclastogenesis and alveolar bone loss.

## Methods

### Ethics statement

The Institutional Animal Care and Use Committee (IACUC) of Augusta University (protocol# 2021-1054) approved all experimental procedures.

### Animal ligature-induced periodontitis

Mature adult (3–6 months) sex-matched males and females C57BL/6J mice or CD73-deficient (B6.129S1-Nt5etm1Lft/J; Strain #:018986) were purchased from the Jackson Laboratory (JAX mice) and subjected to ligature placement on maxillary second molar using silk 5-0 (*n* = 8/group). For ligature placement, mice were anesthesized by intraperitoneal injection of a rodent cocktail consisting of ketamine (100 mg/kg) and xylazine (10 mg/kg). The ligature was left in place in all mice for 8 days of experimental period. The contralateral molar tooth in each mouse was left un-ligated to serve as baseline internal control for alveolar bone loss measurements. At the end of the 8 days, animals were euthanized by carbon dioxide followed by cervical dislocation as secondary method, and hemimaxillae harvested for assessment of alveolar bone loss by microcomputed tomography (micro-CT). Gingival tissues were harvested from the same animals and further processed for RT-qPCR, and primary gingival fibroblasts were primarily cultured for gene expression, protein analysis and wound healing assays. Femurs were extracted from male mice for micro-CT analysis and for differentiation of osteoclasts and subsequent assays *in vitro*. For the micro-CT analysis of femurs and maxillae the investigator was blinded during all analyses. They were housed in cages of five animals maximum per cage in a room with controlled humidity and temperature, and light/dark cycles of 12 h. Approximately 48 animals were used in total.

### Fibroblast cell culture and treatments

Primary murine gingival fibroblasts (mGF) were isolated from healthy mouse gingiva (C57Bl/6J or CD73^−/−^). Briefly, murine cells were isolated and cultured in Dulbecco's Modified Eagle's Medium (DMEM) supplemented with 20% fetal bovine serum and 100 UI/ml penicillin/streptomycin. mGF were cultured in a 5% CO_2_ incubator at 37°C. The growth media was replaced every 2–3 days. Experiments were performed using cells between the 2nd and 8th passages, at approximately 80% confluence as detailed in figure legends. Viable cells were automatically counted using a cell counter and Trypan Blue staining and seeded in uniformity of cell distribution the day before each experiment. Fibroblast characterization was monitored per the expression of Vimentin and Collagen type I (Col1a1) positive staining. For cell treatments, 1 ng/ml mouse recombinant IL-1β (401-ML, R&D systems) was used for the time indicated in each figure legend.

### RNA isolation, cDNA synthesis and quantitative PCR (RT-qPCR)

RNA was extracted from 1 × 10^5^ cells/well with the Invitrogen PureLink RNA Mini Kit (ThermoFisher Scientific) according to the manufacturer's instruction. Reverse transcription was performed using a SuperScript IV VILO Master Mix (ThermoFisher Scientific) to obtain cDNA from Nanodrop read samples. The quantitative PCR was performed using the following inventoried Taqman assays: murine Il1β [Mm00434228_m1]; Il17a [Mm00439618_m1] Cxcl1 [Mm04207460_m1]; Cxcl2 [Mm00436450_m1]; Col1a1[Mm00801666_g1]; Tgfb1 [Mm01178820_m1]; in a 10uL final volume using TaqMan Fast Advanced Master Mix in a StepOne Plus Real-Time PCR system (Applied Biosystems). Relative quantitation of the reference gene (i.e., Actb—Mm02619580_g1) vs. the target gene was performed in duplex reactions and calculated using the comparative Ct (*ΔΔ*Ct) values to generate the RQ for each sample based on the established cycle threshold for each target. Analysis was performed using the StepOne Plus software and Graph Pad Prism.

### Wound healing assay

CytoSelect 24-well wound healing assay (Cell Biolabs, Inc) was used to consistently measure cell migration and/or proliferation for wound closure between mGF derived from WT and CD73-deficient animals. Briefly, mGF were seeded (5 × 10^5^ cells/ml) in the presence of the wound healing inserts for 24 h. After confirmation that a monolayer of cells was formed, the inserts were removed generating a 0.9 mm “wound field”. Cells were left undisturbed and monitored for wound closure after 24 h. mGF were fixed and labeled with DAPI fluorescence to stain cell nuclei. The experiment was done in triplicate for each group and repeated at least 3 times to ensure reproducible results. Pictures were taken using a fluorescence microscope (Keyence BZ-X800 Series) in 4× objective lens and the wound closure was measured per the total fluorescence area using the Fiji software.

### Zymography

Murine gingival fibroblasts were generated as above described. After 24 h of culture, MMP-2 and MMP-9 activity was measured in fibroblast supernatant. In total, 500 ul of mGF supernatant was concentrated by centrifugation for 15 min at 13,000 g using centrifugal filter untis Amicon Ultra Centrifugal Filters (Millipore Sigma). Briefly, 15 ul of supernatant was loaded in 10% Zymogram Plus (Gelatin) Protein Gel to detect MMPs activity. After electrophoresis, gels were incubated for 30 min in 100 ml of 1× Renaturing Buffer. After Renaturing Buffer, the gels were incubated for 30 min in 100 ml of 1× Developing Buffer, then incubated overnight at 37°C in fresh 100 ml of 1× Developing Buffer. Next day, the gels were washed 3× for 5 min each with 100 ml of deionized water. The gels were stained in 40 ml of Page Blue Staining solution for 2 h and washed 3× with deionized water before imaging. Images were acquired using a ChemiDoc Touch Imager (Bio-Rad). The bands on the blots were quantified using the Image Lab Software (Bio-Rad) relative to WT control. Data are representative of three independent experiments in duplicate with similar results.

### Osteoclast differentiation

Osteoclast differentiation was performed as previously described (7). Briefly, bone marrow cells isolated from male femurs were cultured in *α*-MEM supplemented with 1% penicillin/streptomycin and 10% fetal bovine serum (FBS) in the presence of murine Macrophage colony-stimulating factor (M-CSF) (30 ng/ml). After 3 days, adherent cells were treated with M-CSF (30 ng/ml) and murine Receptor activator of nuclear factor kappa beta ligand (RANKL) (10 ng/ml). The medium was changed every other day. After 3 days, cells were stained by tartrate-resistant acid phosphatase (TRAP) staining (Millipore Sigma) and images were captured using the BZ-X810 microscopy (Keyence) using 10× objective lens. Cells were treated and collected according to each set of experiments. TRAP + cells containing three or more nuclei/cell were quantified. Total mature osteoclasts were counted and represented as TRAP + cells/well.

### Resorption pit assay

Murine adherent cells were seeded onto 96-well hydroxyapatite-coated plates (OsteoAssay-Corning) using osteoclastogenic media, as described in our osteoclast differentiation protocol. After 4 days, the cells were removed, and the extent of bone resorption was quantified by measuring the size and number of resorption pits formed by osteoclasts on the bone substrate. Image analysis software (ImageJ) was employed to calculate the pit area and resorption activity after acquiring images in a Keyence BZ-X800 Series microscope using a 10× objective lens. The investigator was blinded during all analyses.

### Western blot

For Western blot (WB) analyses, cold RIPA buffer (ThermoFisher) was applied to extract total protein from 1 × 10^6^ cells/mL in 24 well plates. The extracted proteins were measured using the BCA protein assay reagent kit (Pierce Protein Biology). An equal amount of total protein (10 μg of protein/lane) was then resolved with a 5%–12% SDS-PAGE gel and electrotransferred onto a polyvinylidenedifluoride (PVDF) membrane (Bio-Rad) in a fast-transfer mode. The blots were developed using an enhanced chemiluminescence (ECL) detection kit (Bio-Rad) and visualized using a ChemiDoc Touch Imager (Bio-Rad). The bands on the blots were quantified using the ImageLab Software (Bio-Rad) and normalized for the loading control for densitometry analysis. Data are representative of three independent experiments in duplicate with similar results. The original uncropped images of all WB figures presented are provided in Supplementary Figure S1.

### Antibodies

WB experiments were performed using the following antibodies with respective dilutions: Antibodies from Abcam: Total OXPHOS Rodent WB cocktail (1:1,000, ab110413); Cathepsin K (1:3,000 #ab207086); Goat Anti-rabbit IgG (HRP) (1:25,000 #ab97051); Antibodies from Cell Signaling: Catalase (1:1,000, #14097); HRP Conjugate *β*-Tubulin (9F3) (1:1,000, #5346); HRP conjugate H3 (1:1,000 # 12648), HRP conjugate *β*-Actin (1:1,000 # 12620); HRP Conjugate GAPDH (1:1,000, #3683); CD73 (1:1,000, #PA5-85958, Invitrogen); ATP6V0D2 (1:1,000 #NBP1-54595, Novus Biologicals).

### Hydrogen peroxide (H_2_o_2_) measurement

H_2_O_2_ levels were measured using the ROS-GLO H_2_O_2_ assay (Promega), following the manufacturer's instructions. Briefly, osteoclasts were seeded in opaque 96-well plates as described in our osteoclast differentiation protocol. On day 3 of RANKL differentiation, the H_2_O_2_ substrate solution was added to the cell culture at a final concentration of 25*μ*M and incubated for 6 h. After this period, the cells were incubated at room temperature for 20 min with 100 μl of detection solution containing luciferin. Next, the relative luminescence units were recorded using a luminescence plate reader (SynergyH1, BioteK).

### Assessment of mitochondrial membrane potential

Mitochondrial membrane potential was assessed with Cell Meter™ JC-10 Mitochondrion Membrane Potential Assay Kit (AAT Bioquest) following the manufacturer's recommendations. Briefly, cells were seeded onto 96 well black/clear bottom plate and cultured as described in our osteoclast differentiation protocol. On day 3, the JC-10 dye solution was added for 30 min at 37°C, 5% CO_2_. The fluorescent intensities for both J-aggregates and monomeric forms of JC-10 were analyzed by a plate reader (SynergyH1, BioTek) at Ex/Em = 490/525 and 540/590 nm.

### Human sample data

Transcript (mRNA) levels of CD73 were derived from stored peripheral blood mononuclear cell (PBMCs) cDNA from Grade C molar/incisor pattern vs. healthy individuals (*n* = 30/group) as previously described (8). All research subjects were enrolled in the previous study (8) after providing written informed consent approved by the Institutional Review Board (IRB) of the University of Florida in Gainesville (IRB#201400349) and University of Kentucky (IRB#44933). The cohort re-examined in this study was part of a registered clinical trial (Clinicaltrials.gov #NCT01330719).

### Statistical analysis

Statistical analysis was conducted for three independent experiments using the GraphPad Prism v9 software (GraphPad, San Diego, CA, USA) using ANOVA followed by multiple comparison tests. Data are presented as mean ± S.D. The cell number per well was chosen based on cell density optimization experiments for the specific assay. The significance level of *p* is indicated in each graph and in figure legends (*p < 0.05; **p < 0.01; ***p < 0.001; ****p < 0.0001).

## Results

### CD73 deficiency leads to increased gingival inflammatory markers in experimental periodontitis and hyper-inflammatory response with defective healing capacity of gingival fibroblasts

**Figure 1:**
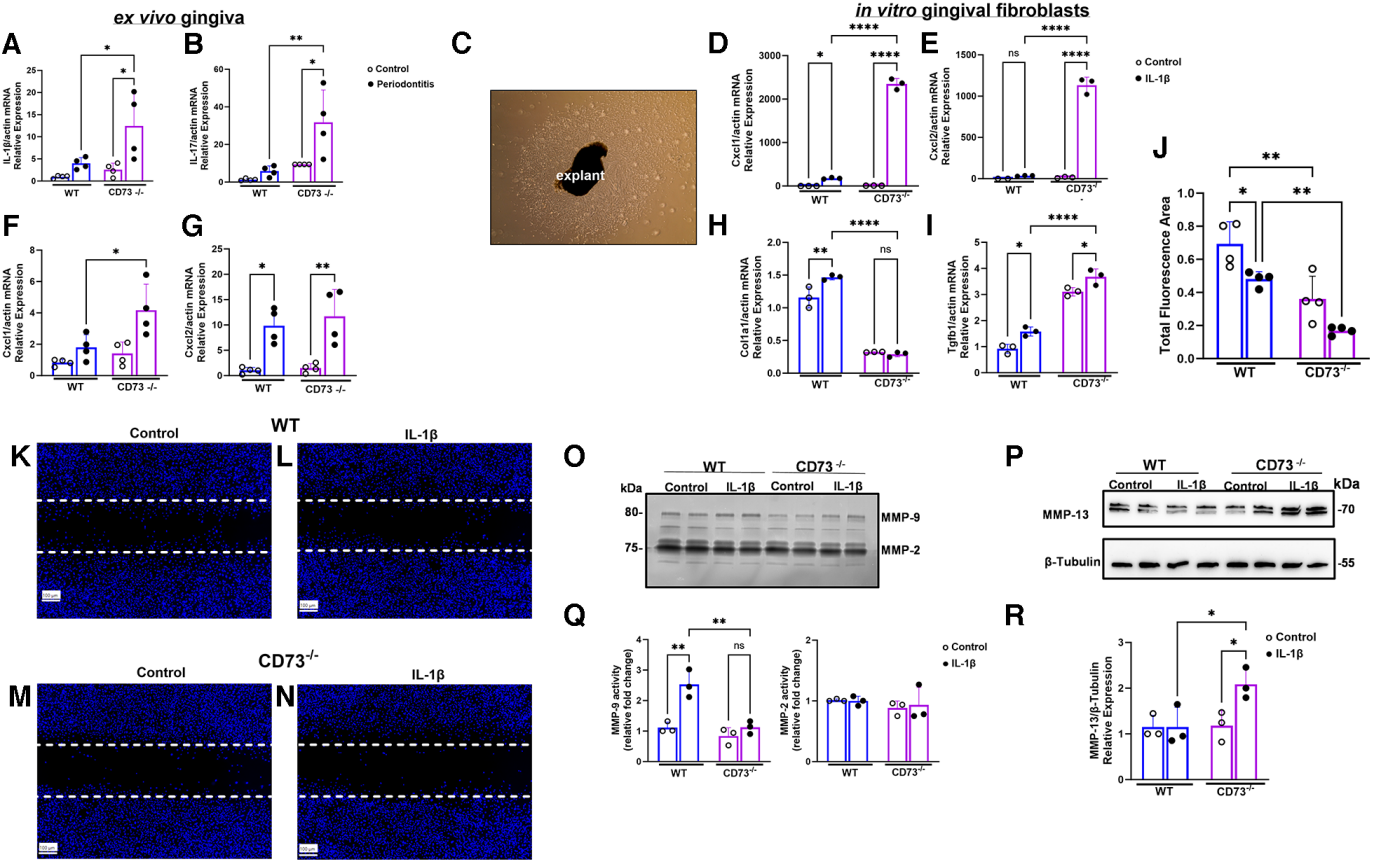
Cd73 deficiency leads to increased gingival inflammatory markers in experimental periodontitis, hyper-inflammatory response with defective healing capacity and increased MMP-13 in gingival fibroblasts. The gingival mRNA expression of murine (**A**) Interleukin-1 beta (IL)-1β, (**B**) IL-17, (**F**) Cxcl1, and (**G**) Cxcl2 relative to β-actin as a reference gene were measured by RT-qPCR in WT and CD73^−/−^ animals comparing control (unligated side) vs. periodontitis (ligated side). (**C**) Primary murine gingival fibroblasts (mGF) explants depicts primary cells after 8 days of culture. The mGF mRNA expression of (**D**) Cxcl1 (**E**) Cxcl2 (**H**) Collagen-1(Col1a1), and (**I**) Tgf-β (Tgfb1) relative to β-actin as a reference gene by RT-qPCR after 24 h of murine IL-1β stimulation (1 ng/ml). (**J**) Total Fluorescence area of wound closure for WT and CD73-deficient fibroblasts submitted to wound healing assay. Representative images of wound healing assay demonstrating the cell invasion into the cell-free region (outlined) is impaired in CD73-deficient cells. Images depicting (**K**) WT control or (**L**) WT with IL-1β, (**M**) CD73^−/−^ control and (**N**) CD73^−/−^ with IL-1β. Cell nuclei were stained with DAPI (4′,6-diamidino-2-phenylindole) blue-fluorescent DNA stain to determine total fluorescence area representing wound closure after 24 h. (**O**) MMP-9 and MMP-2 activity by zymography image and respective (**Q**) densitometric analysis of mGF supernatants derived from WT or CD73^−/−^ -animals with or without IL-1β stimulation (1 ng/ml) for 24 h. (**P**) MMP-13 protein expression by immunoblot of mGF extracts derived from WT or CD73^−/−^ -animals with or without IL-1β stimulation (1 ng/ml) for 24 h. (**R**) MMP-13 densitometry analysis compared to *β*-Tubulin as loading control. Data are presented as mean ± S.D. (*p < 0.05; **p < 0.01; ***p < 0.001; ****p < 0.0001). Scale bars: 100 µm.

In a previous study, we have shown CD73-dependent adenosine dampens the inflammatory-associated phenotype of human gingival fibroblasts typified by hyper secretion of CXCL8 chemokine induced by IL-1β (9). In the present study, the local inflammatory response in murine gingiva was determined *ex vivo* by measuring mRNA levels of key inflammatory markers in the pathogenesis of periodontitis using ligature-induced periodontitis model as previously described (10) and previously performed by our group (11). We detected a significant increase in mRNA levels of Interleukin (IL)-1β, IL-17 and the chemokines Cxcl1 and Cxcl2 which are important neutrophil chemoattractants (corresponding to Cxcl8 in humans) in gingival tissue derived from CD73-deficient animals with periodontitis when compared to the WT (Figures 1A,B,F,G). We then isolated the primary fibroblasts from explants of murine gingiva (Figure 1C) and challenged them *in vitro* with murine IL-1β (1 ng/ml) to see the response of the gingival dominant cells in periodontium. Inflammatory-associated state of gingival fibroblasts induced by IL-1β revealed significantly higher expression of Cxcl1 and Cxcl2 in CD73-deficient fibroblasts with decreased Col1a1 and increased Tgf-β1 (Figure 1D,E,H,I). Even though the implications of Tgf-β1 on fibroblast proliferation are inconsistent in existing literature and may typically be context-dependent of other interacting mediators (12) many studies have reported antiproliferative effects (13, 14). A previous study has demonstrated loss of CD73 being context determinant for increased Tgf-β (15), which in our model combined with decreased Col1a1 could impact healing outcome. We also detected decreased wound healing capacity of CD73-deficient fibroblasts, using the wound healing assay *in vitro* which resulted in significantly lower area covered by CD73-deficient fibroblasts both in the presence or absence of IL-1β (Figure 1J). Representative images of decreased closure of the wounded area (dashed lines) are shown in Figure 1K–N. Further analyzed was the activity of gelatinases MMP-2 and MMP-9 (Figure 1O) and the protein expression of collagenase MMP-13 (Figure 1P), since previous research has shown the importance of MMPs in wound healing (16). Previous studies have demonstrated the relevance of MMP-2 and MMP-9 in periodontal diseases (17) and increased MMP-13 activity in both murine model of periodontitis (18) and human periodontitis (19). In our model, densitometry analysis revealed that constitutive activity of MMP-2 was not different between WT or CD73-deficient cells, but MMP-9 activity was significantly lower in fibroblasts derived from CD73^−/−^ mice (Figure 1Q). The full functional implications of MMP-9 in CD73-deficient fibroblasts remain to be defined. However, MMP-9 seems to play a key role in modulating tissue reorganization mediated through fibroblast contractile activity (20) and mice that lack MMP-9 display delayed wound healing (21), consistent with our results. Furthermore, we detected significantly higher MMP-13 in CD73-derived fibroblasts challenged with IL-1β (Figure 1R). These results indicate the consequences of CD73 deficiency in stromal fibroblasts which presented a hyper-inflammatory response, defective healing capacity and increased MMP-13 expression in the context of periodontal inflammation.

### CD73 deficiency leads to bone loss and aggravate ligature-induced alveolar bone loss

**Figure 2:**
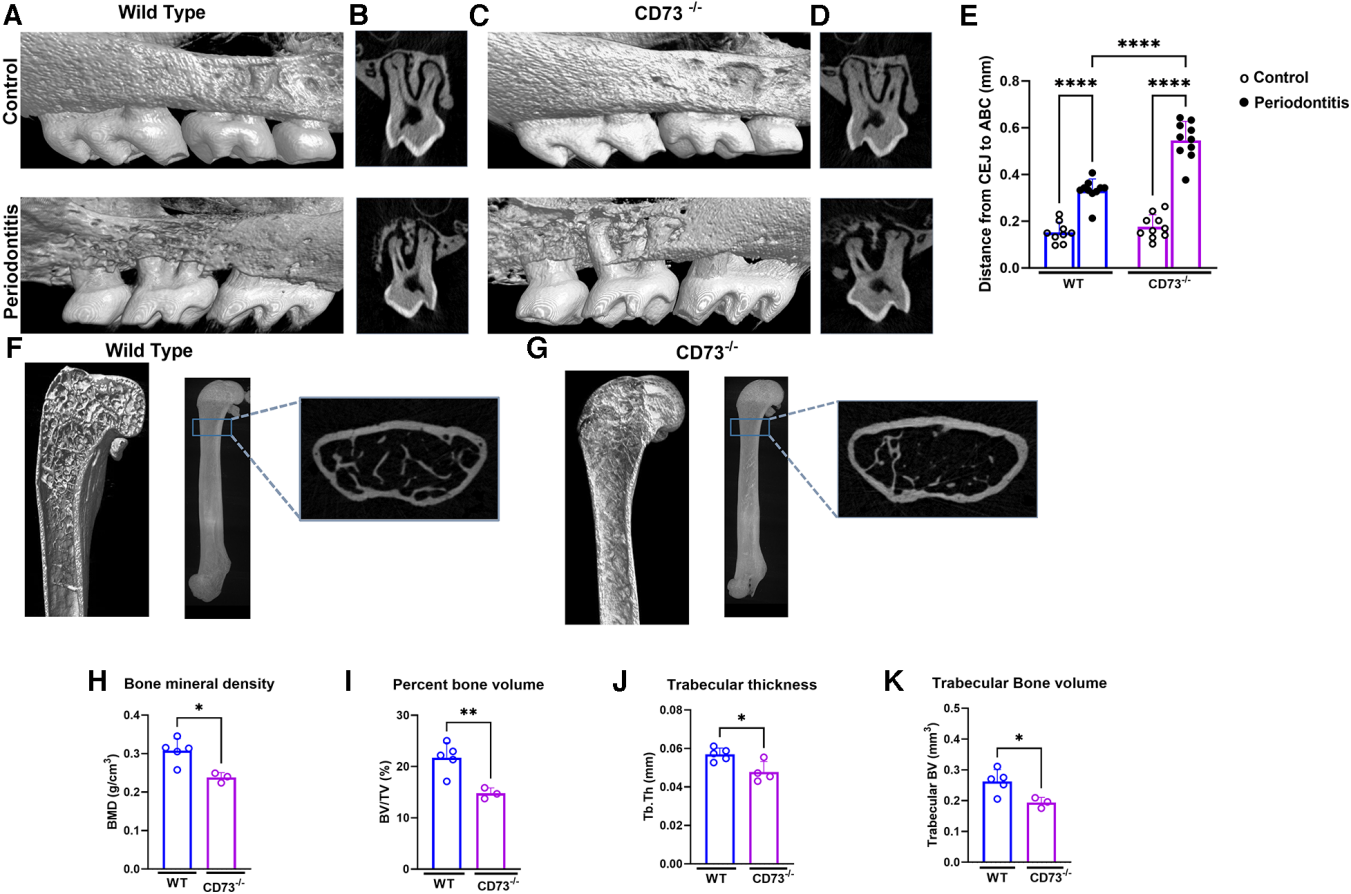
The lack of CD73 leads to increased bone loss. Representative 3-dimensional images from hemi-maxillae (upper panels) unligated control (left side) vs. (lower panels) periodontitis (right side) from (**A**) WT and (**C**) CD73^−/−^ mice (*n* = 8 mice/group). (**B,D**) show representative 2-dimensional images of ligated maxillary 2nd molar in control vs. periodontitis for each experimental group. (**E**) Alveolar bone loss was measured per the distance from cemento-enamel junction (CEJ) to alveolar bone crest (ABC) in mm in control (open dots) and periodontitis (closed dots) comparing WT and CD73^−/−^ animals. Micro-computed tomography data analysis of long bones of (**F**) WT or (**G**) CD73^−/−^ mice, including (**H**) bone mineral density (BMD), (**I**) percent bone volume (BV/TV), (**J**) trabecular thickness (Tb. Th) and (**K**) trabecular bone volume (mm^3^) *n* = 3-5 mice/group. Representative 3-dimensional longitudinal and 2-dimensional cross-sectional views from femurs. Data are presented as mean ± S.D. (*p < 0.05; **p < 0.01; ***p < 0.001; ****p < 0.0001).

Due to recent studies revealing that MMP-13 produced by mesenchymal cells plays a role in the loss of bone mass (22) and because bone resorption is the hallmark of periodontitis, we then decided to investigate the role of CD73 in bone loss in our experimental model of ligature-induced periodontitis. We ligated the upper right second molar according to our previous work (11) and left the upper left second molar unligated to serve as an internal control for alveolar bone loss. After 8 days of ligature-induced periodontitis, we assessed the alveolar bone loss by micro-CT. We confirmed significant bone loss in the ligated site (right side) vs. the unligated control site (left side) after 8 days of disease induction (Figures 2A–D). Our results revealed a significantly greater alveolar bone loss reflected by the distance from the cemento-enamel junction to the alveolar bone crest in mm in CD73-deficient animals when compared to WT (Figure 2E).

Since adenosine signaling has been investigated previously in bone metabolism and the signaling via adenosine receptor A2bR has been described to play an important role in bone homeostasis (23) positively regulating osteoblast differentiation (3), we decided to check the long bone phenotype of CD73-deficient animals. The phenotype of aggravated bone loss in CD73-deficient animals was also seen in long bones (Figures 2F,G). When we compared the long bones (femurs) of the C57Bl/6J wild-type (WT) vs. CD73 knockout mice (CD73^−/−^), we detected a significant lower bone mineral density, percent bone volume, trabecular bone volume and trabecular thickness (Figures 2H–K) as also shown in the cross section slices presented in Figures 2F,G. These data confirmed that in the absence of functional CD73, bone loss was increased which then prompted us to investigate specifically the phenotype of osteoclasts which are key players controlling bone resorption.

### The lack of CD73 leads to increased osteoclastogenesis *in vitro*

**Figure 3:**
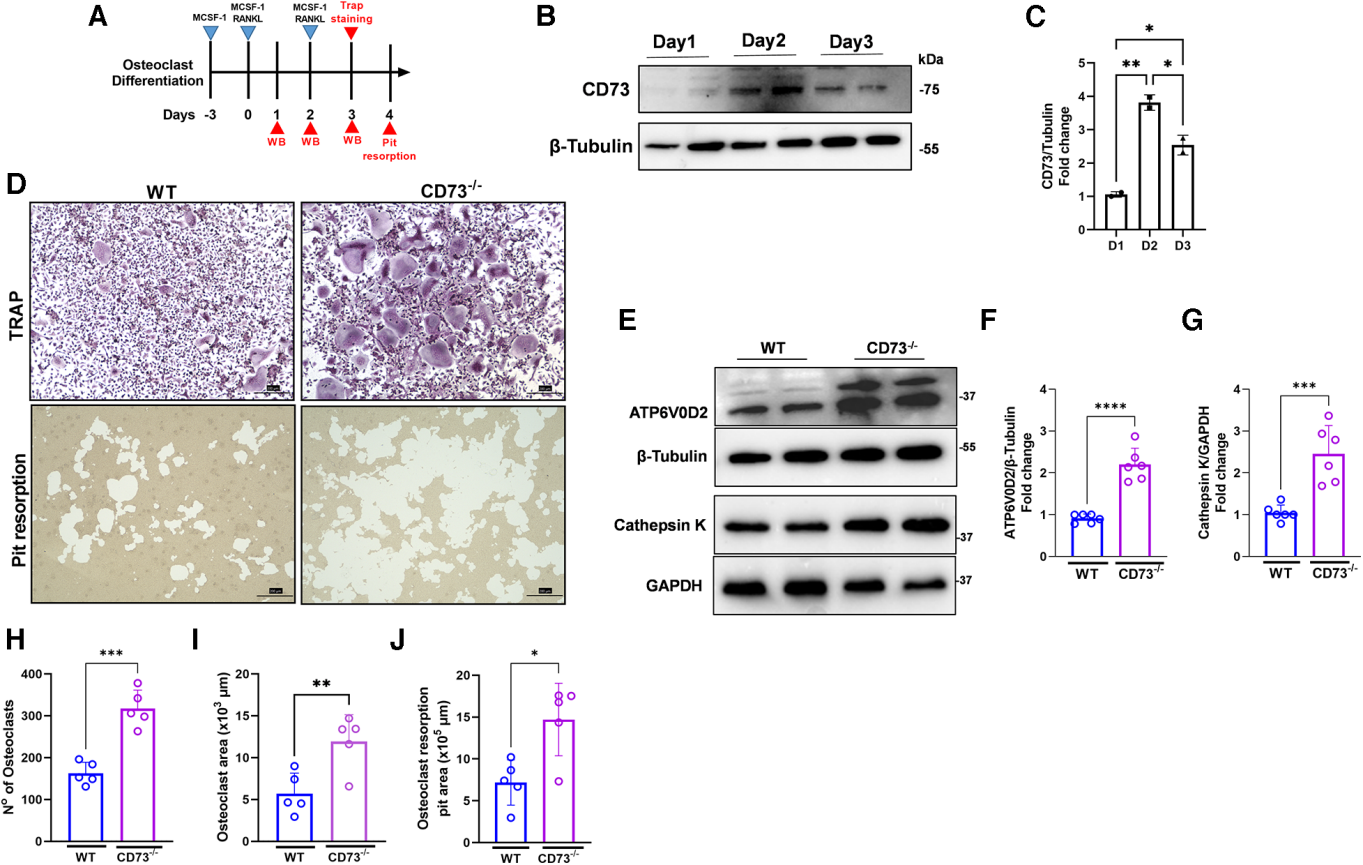
The lack of CD73 leads to increased osteoclastogenesis *in vitro.* (**A**) Osteoclast differentiation model in the presence of M-CSF and RANKL. (**B**) Protein expression with the respective (**C**) densitometry analysis of CD73 relative to the loading control β-Tubulin during differentiation of osteoclasts (1–3 days). (**D**) Identification of activate osteoclasts using TRAP positive staining (upper panels) and Pit resorption assay (lower panels) between WT (left panels) and CD73^−/−^ (right panels). Protein expression of (**E**) ATP6V0D2 and Cathepsin K in osteoclasts derived from WT or CD73^−/−^ animals with the respective (**F, G**) densitometry analysis showing fold change relative to *β*-tubulin or GAPDH as loading control. Measurements of (**H**) number of osteoclasts, (**I**) Osteoclast area (x 10^3^ µm), and (**J**) Osteoclast resorption pit area (x 10^5 ^µm). Data are presented as mean ± S.D. (*p < 0.05; **p < 0.01; ***p < 0.001; ****p < 0.0001).

We then investigated the role of CD73 in the differentiation and activation of osteoclasts. Our osteoclast differentiation model using a combination of M-CSF and RANKL is shown in Figure 3A. We detected a peak of CD73 expression on day 2 of osteoclast differentiation (Figure 3B), and as cells progressed towards a more differentiated state with 3 days, the CD73 expression decreased in WT-derived cells (Figures 3B,C). When we compared the osteoclast differentiation between WT and CD73-deficient cells, we saw a significantly higher osteoclast differentiation by Tartrate-resistant acid phosphatase (TRAP)-positive staining in CD73-deficient cells (Figure 3D). Both the number and size of osteoclasts were increased with the lack of functional CD73 (Figures 3H,I). CD73-deficient osteoclasts also resorbed more bone *in vitro,* as detected per the higher pit resorption in CD73^−/−^ cells compared to WT (Figure 3D—lower panels, J).

Bone resorption relies on the extracellular acidification function of vacuolar (V-) ATPase proton pump(s) present in the plasma membrane of osteoclasts. Because ATP6V0D2, an isoform of the d subunit in the V-ATPase has been shown to be highly expressed in mature osteoclasts, being a regulator of cell fusion in osteoclast differentiation (24), we investigated the protein expression of ATP6V0D2 in osteoclast extracts. We have also investigated the expression of Cathepsin K, which is a lysosomal cysteine protease whose highest expression is found in osteoclasts being the active form localized in the ruffled borders of osteoclasts (25). The enzymes V-ATPaseD2 (ATP6V0D2) (Figures 3E,F) and Cathepsin K (Figures 3E,G) were significantly increased in CD73-deficient osteoclasts confirming increased bone-resorbing activity of osteoclasts in the lack of CD73.

### CD73 deficiency leads to increased osteoclastogenesis through higher oxidative phosphorylation

**Figure 4:**
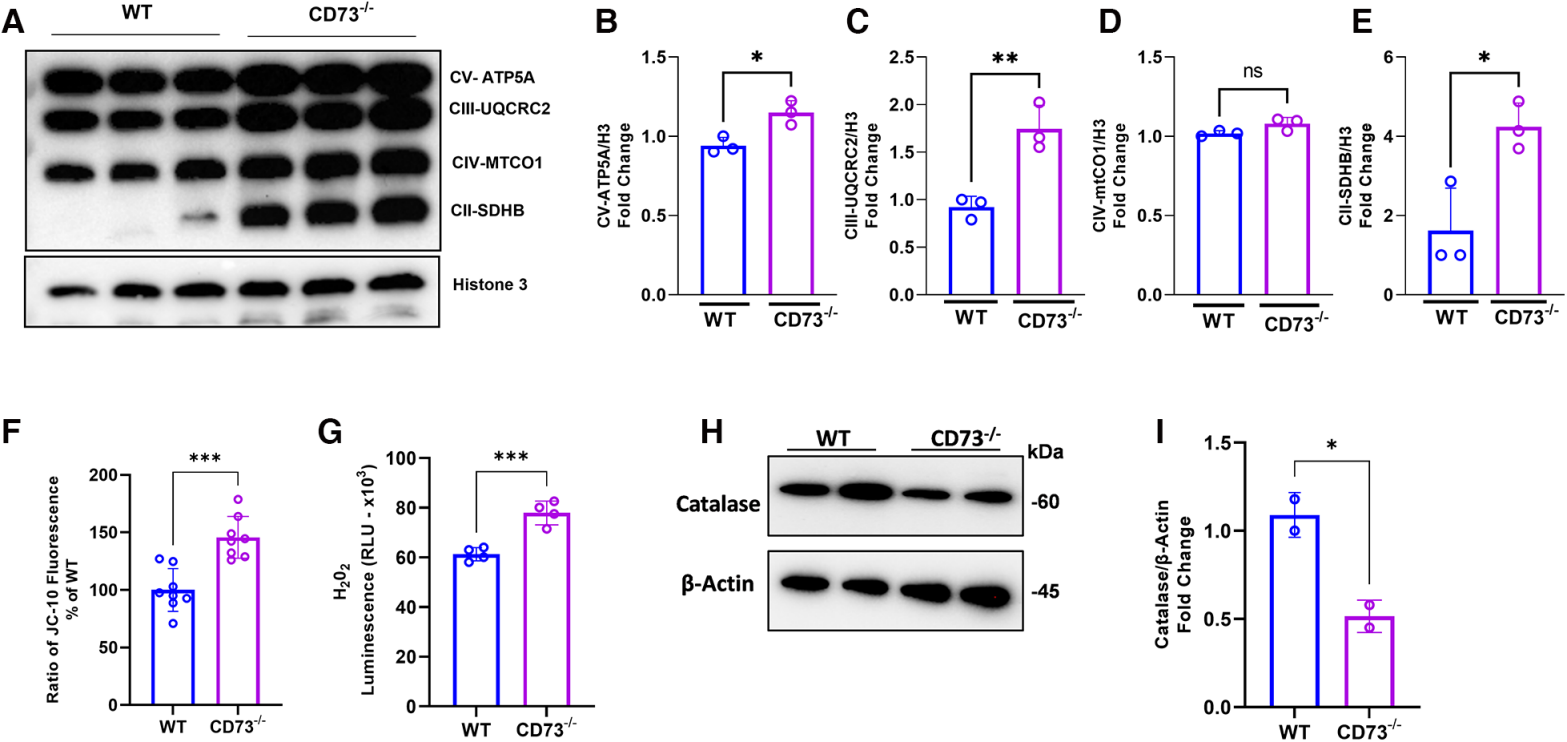
The lack of CD73 leads to increased oxidative phosphorylation, increased mitochondrial membrane potential and imbalance between H_2_O_2_ and catalase levels. Increased mitochondrial oxidative phosphorylation detected through (**A**) increased expression of OXPHOS protein complexes in CD73^−/−^ -derived osteoclasts compared to WT-derived osteoclasts. Densitometry analysis of subunits of (**B**) Complex V—ATP5A, (**C**) Complex III—UQCRC2, (**D**) Complex IV—MTCO1, (**E**) Complex II-SDHB. Increased mitochondrial membrane potential represented by (**F**) the ratio of JC-10 Fluorescence in CD73^−/−^ -derived osteoclasts relative to WT cells. (**G**) Intracellular H_2_O_2_ accumulation measured by luminescence in WT and CD73^−/−^-derived osteoclasts. (**H**) Catalase protein expression in WT and CD73^−/−^-derived osteoclasts with the respective (**I**) densitometry analysis relative to *β*-actin as loading control. Data are presented as mean ± S.D. (*p < 0.05; **p < 0.01; ***p < 0.001, ns = not significant).

Our previous work showed that adenosine boosts mitochondrial health in stromal cells through mitochondrial biogenesis, leading to decreased inflammation (26). Accordingly, we assessed how mitochondrial metabolism of osteoclasts is impacted by lack of adenosine resulting from CD73 deficiency. Mitochondrial oxidative phosphorylation (OXPHOS) is the main bioenergetic source for human osteoclast formation (27). Our hypothesis is that the increased osteoclastogenesis detected in CD73-deficient cells is driven by increased expression of the OXPHOS complexes in CD73-deficient osteoclasts. We confirmed oxidative phosphorylation was increased as evidenced by significant upregulation of mitochondrial complexes of the electron transport chain (ETC), such as subunits of complex V, III and II in CD73-deficient osteoclasts compared to WT cells (Figures 4A–E). The mitochondrial membrane potential was also increased in CD73-deficient cells (Figure 4F), which supports our hypothesis that the higher osteoclastogenesis in CD73-deficient cells was associated with higher mitochondrial activity in these cells.

The OXPHOS is not only responsible for producing high-energy phosphates, such as ATP, but are also involved in various cellular processes, including generating reactive oxygen species (ROS) (6, 28). Osteoclasts from CD73-deficient mice exhibited higher H_2_O_2_ levels during RANKL-induced differentiation as compared with WT-derived cells (Figure 4G). Because we detected high levels of ETC proteins and ROS, we examined the expression of the enzyme catalase, a crucial antioxidant enzyme responsible for breaking down hydrogen peroxide into water and oxygen. As expected, catalase was decreased in CD73-deficient osteoclasts (Figures 4H,I) which corroborates the notion that the lack of CD73 showed to accumulate functional osteoclasts due to increased OXPHOS leading to the accumulation of H_2_O_2_ and lack of clearance of ROS, because of decreased catalase.

### Decreased CD73 level is associated with severe grade C molar-incisor periodontitis

**Figure 5:**
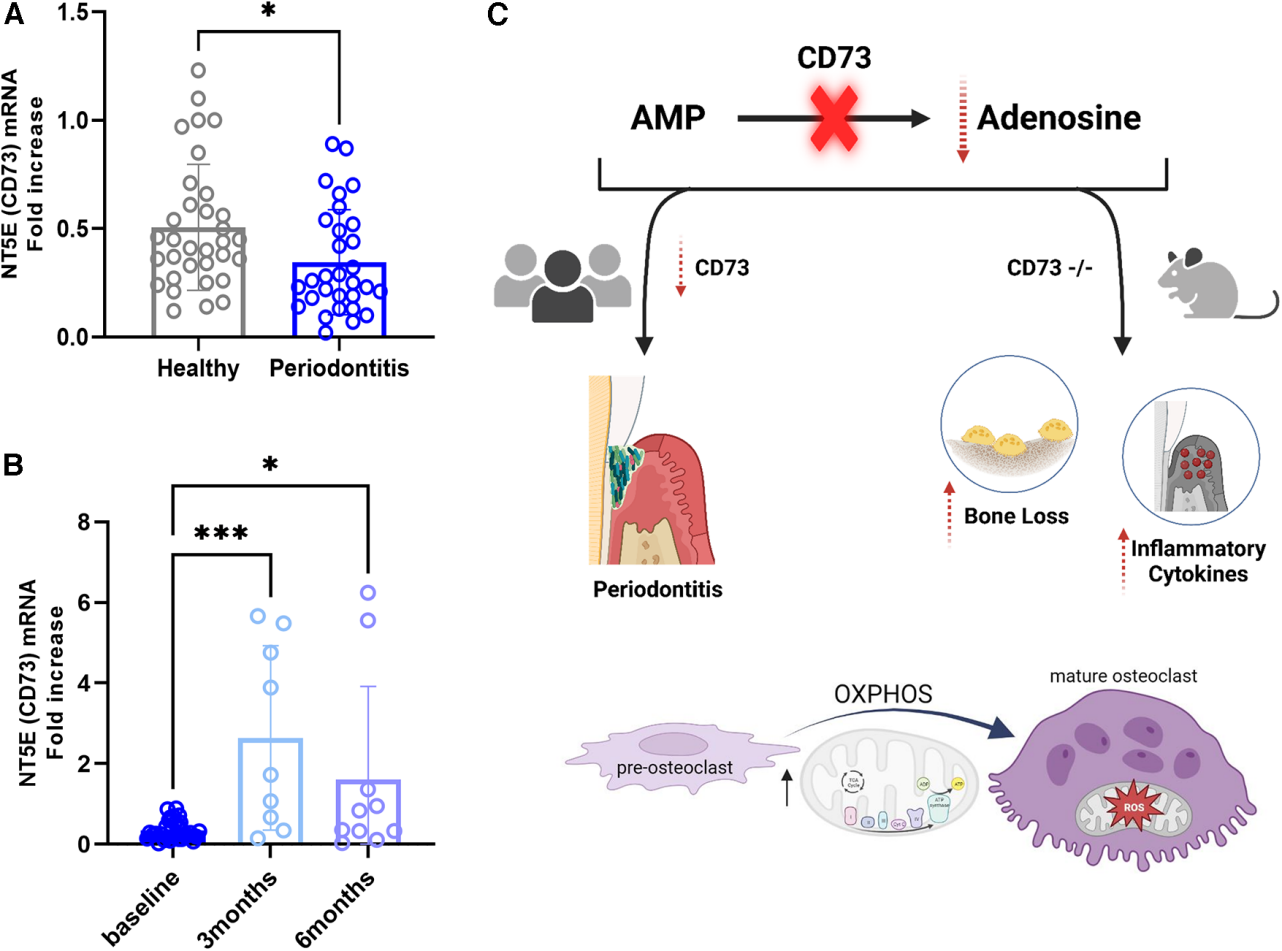
Cd73 ectonucleotidase is decreased in severe periodontitis and mRNA levels are rescued after 3 or 6 months of periodontal treatment. Human correlative studies showing (**A**) NT5E (CD73) mRNA levels in healthy and periodontitis samples (*n* = 30/group) classified as Grade C/MIP. (**B**) increased CD73 mRNA in periodontitis samples after 3 or 6 months post-periodontal therapy of scaling and root planning (*n* = 9-10/group). (**C**) Summary of our findings showing that CD73 deficiency is associated with severe periodontitis in humans and the lack of CD73 leads to bone loss characterized by dysregulated osteoclast energy metabolism and gingival hyper-inflammatory cytokine expression in murine model of periodontitis. Data are presented as mean ± S.D. (*p < 0.05; ***p < 0.001).

Because we wanted to assess the relevance of CD73 in regards to the severity of periodontitis, in this study we assessed the mRNA levels of CD73 in PBMCs from patients affected with a severe manifestation of the disease classified currently as Grade C molar-incisor pattern (C/MIP) from an existing cohort of patients (8). We detected a significant reduction in CD73 levels in periodontitis Grade C/MIP compared to healthy individuals age matched (5–25 years old) (Figure 5A). We also compared the baseline levels with the CD73 expression after 3 months or 6 months post non-surgical periodontal treatment [scaling and root planing (SRP)] (Figure 5B). Our results demonstrated increased CD73 mRNA after 3 months or 6 months of SRP compared to baseline (Figure 5B) which confirms the relevance of the CD73 ectonucleotidase to the pathogenesis of periodontitis, specially in a clinical pattern of severe, rapid rate of bone destruction and early onset form of disease. A summary of our findings is provided in the schematic drawing in Figure 5C.

## Discussion

In the present study, we demonstrated for the first time that the ectonucleotidase CD73 [also known as 5′-nucleotidase ecto (NT5E)] has a protective role in periodontitis. By combining *in vivo* ligature-induced periodontitis mouse model in CD73-deficient animals with *ex vivo* and *in vitro* cell analysis comparing WT and CD73-deficient cells, we showed higher levels of pro-inflammatory markers in local gingiva with decreased healing capacity and higher levels of MMP-13 in CD73-deficient stromal fibroblasts, revealing a hyper-inflammatory profile in gingival fibroblasts as a consequence of the lack of CD73.

We have previously shown CD73-dependent adenosine was implied in decreased inflammatory chemokine production by human gingival fibroblasts through adenosine receptors (9), emphasizing its role as a negative regulator of gingival inflammation. Recently, we demonstrated the mitochondrial metabolism and generation of new mitochondria was mechanistically involved in the anti-inflammatory effects observed upon extracellular adenosine exposure in IL-1β-stimulated gingival fibroblasts (26). In physiological conditions, adenosine produced by CD73 enzymatic activity protects tissues against excessive inflammation. Therefore, our results obtained in the present study are consistent with our previous reports and with the notion that the absence of CD73 leads to a dysregulation in endogenous modulated inflammation, leading to a hyper-inflammatory response in gingival stromal cells due to the lack of adenosine-mediated homeostasis.

There is a large body of evidence showing CD73 expression dictates reparative properties and anti-inflammatory activity in models of cardiac repair in different cells such as mesenchymal stem cells (29) or regulatory *T*-cells (30, 31). It is also noteworthy that CD73 has been recognized as a surface marker for cultured fibroblasts expressing mesenchymal stem cell-associated surface markers (32). In humans, gingival fibroblast secretome accelerates wound healing as inflammation within the wound bed was decreased with gingival fibroblast or the conditioned media while collagen deposition was improved (33). Here, we observed the lack of CD73 resulted in worse healing outcome *in vitro* and decreased transcription for collagen type I in mGF, which could also be due to the alteration in stemness properties of these cells. The exact mechanism involving the role of CD73 in gingival fibroblast healing response is currently under further investigation in our lab. We also investigated MMP-2, MMP-9 and MMP-13 levels and interestingly, MMP-13 levels were significantly higher in CD73-derived mGF challenged with IL-1β. Previous studies have shown the correlation of MMP-13 with increase in severity of inflammation in human periodontitis (19) and different models of experimental periodontitis (34) and that inhibition of MMP-13 reduced inflammatory bone resorption *in vivo* (18). Furthermore, we find it intriguing that mesenchymal-derived MMP-13 can also be responsible for increase in osteoclast number and activity (22). Thus, although speculative at this point it is possible that CD73 ectonucleotidase regulates MMP-13 levels in stromal gingival compartment which in turn can influence osteoclast activity.

The lack of CD73 not only showed a more destructive bone phenotype in long bones but also an increased alveolar bone loss in experimental periodontitis with higher osteoclast differentiation and activity. Increased RANKL-induced osteoclastogenesis *in vitro* was characterized by a significantly upregulation of metabolic activity of CD73-deficient osteoclasts via oxidative phosphorylation. The loss of function of CD73 lead to decreased catalase-1, which is one of the antioxidant enzymes functioning to prevent H_2_O_2_ accumulation and cell damage in osteoclasts (28). An increase in oxidative phosphorylation in osteoclasts refers to an enhanced metabolic process occurring within these bone-resorbing cells. Therefore, it indicates that these cells are experiencing higher energy demands to carry out bone resorption (35). This energy-intensive process requires ATP to fuel the enzymatic reactions involved in breaking down the bone (36, 37) and are indicative of development of mature osteoclasts, confirming pathways previously implicated in osteoclast biology (35). In other words, the CD73-dependent maximization of mitochondrial activity we observed in our model was followed by the optimization of osteoclast multinucleation, resulting in more bone resorption. Cell-cell fusion and multinucleation were previously described to enhance the mitochondrial activity required for resorptive activity in osteoclasts (38). Here we also show the lack of CD73 leads to intracellular H_2_O_2_ accumulation, higher mitochondrial membrane potential and higher expression of the main subunits of the ETC, which have all been reported to be critical for the differentiation and survival of bone resorbing osteoclasts (27, 39). Notably, the fact that mitochondria-related enzyme catalase was significantly lower in CD73-deficient osteoclasts, leading to the predominance of oxidative stress confirm our hypothesis that excessive osteoclast mitochondrial function is involved in the increased osteoclastogenesis in our model.

The role of OXPHOS as a predominant bioenergetic pathway during osteoclast differentiation has been previously investigated through various approaches, including inhibition of ETC complexes and disruption of the mitochondrial network. Lack of Ndusf4 (Complex I subunit) or blockade of Complex I activity as a model of mitochondrial dysfunction in precursor cells inhibits osteoclast differentiation and the formation of resorption pits *in vitro* (39). Inducing mitochondrial dysfunction by a double conditional knockout model deleting mitofusins (Mfn1 and Mfn2) in osteoclast precursors, knockdown of Dynamin-related protein 1(Drp1), or deficiency of Opatic atrophy 1 (Opa1), which are essential proteins involved in mitochondrial fusion and fission also leads to decreased osteoclastogenesis by impairing signaling to NFATc1, a master transcription factor for osteoclast differentiation (40–42). Ultimately, glucose metabolism, mitochondrial biogenesis, and oxidative respiration were identified as essential pathways that alter osteoclast differentiation and resorptive activity (43).

We revealed a previously unappreciated relationship between lower CD73 levels and severe manifestation of periodontitis (in the form of Grade C/MIP), at least at the mRNA level in young individuals since the present cohort involved ages 5–25 years old. Surprisingly, CD73 transcript levels were upregulated at 3 months and 6 months of follow up after scaling and root planning, which could indicate CD73 as a potential candidate biomarker for treatment monitoring or perhaps a prognosis indicator associated with evolution of the disease. Of course, here we recognize the limitation of the sample size and the nature of the data obtained at the mRNA level, which prompts the need of more research to measure accurately the prognostic and predictive value of CD73 expression in periodontal pathogenesis, including age-matched cohort for mild to moderate manifestation of periodontitis.

Although the major damage occurs in periodontal tissues, peripheral blood leukocytes, as the source of local leukocytes, can contribute to periodontitis by influencing the destructive host immune response (44). Recent report on transcriptome changes of PBMCs in periodontitis at single-cell resolution sustain the relevance and appropriateness of analyzing this type of sample. Single-cell RNA seq data revealed the systemic immunological effects of periodontitis and identified periodontitis-specific predictors of inflammation. In addition, based on the immune pathways that were responsive to therapy, nonresponsive pathways to treatment that can increase the risk of comorbidity were found (45). Given the limitations of the data presented in our study, we believe it is relevant to explore the CD73 levels in cDNA derived from PBMCs. There is an increasing interest in establishing peripheral/systemic biomarkers to enhance precision/personalized periodontal medicine for diagnosis, treatment-response prediction and prognosis. Our findings are summarized in Figure 5C.

## Conclusions

In summary, our data reveal a key protective role for the ectonucleotidase CD73 in the pathogenesis of periodontitis. We showed the lack of CD73 activity leads to increased gingival inflammatory markers and an inflammation-associated gingival fibroblast state with an impaired wound healing profile. We also revealed CD73 deficiency leads to higher bone loss, increased ligature-induced alveolar bone loss, and more osteoclastogenesis characterized by increased mitochondrial oxidative phosphorylation and dysregulation of H_2_O_2_/catalase levels. Our *in vivo* and *in vitro* data is in agreement with our clinical correlative studies, which unveiled decreased CD73 levels associated with severe periodontitis characterized by rapid rate of bone destruction. Our data reveal CD73 as a new potential biomarker, which can be considered to track excessive osteoclast activity in periodontitis, uncovering novel avenues for future preventive strategies or early therapeutic intervention in inflammatory and osteolytic diseases.

## Data Availability

The original contributions presented in the study are included in the article/**Supplementary Material**, further inquiries can be directed to the corresponding author.
