# Supplementary material for: The protective role of CD73 in periodontitis: preventing hyper-inflammatory fibroblasts and driving osteoclast energy metabolism
Source: Front Oral Health. 2023 Dec 13;4:1308657. doi: 10.3389/froh.2023.1308657 (PMC10751373; doi:10.3389/froh.2023.1308657)
Supplement: Supplementary file 1 [file Image1.pdf]

Supplementary Material

The protective role of CD73 in periodontitis: preventing hyper-inflammatory fibroblasts and driving osteoclast energy metabolism

Ramos-Junior ES[1], Dawson S[1], Ryan W[1], Clinebell B[1], Serrano-Lopez R[1], Russell M[1], Brumbaugh R[1], Zhong R[2], Goncalves J[3], Shaddox LM[3], Cutler CW[4], Morandini AC[1,4,*]

***Corresponding Author:**

AC Morandini, Department of Oral Biology and Diagnostic Sciences,

Mailing address: 1120 15th Street, CB-2404F

Augusta, GA 30912

(706) 721-2582

amorandini@augusta.edu

Figure 1P

MMP-13

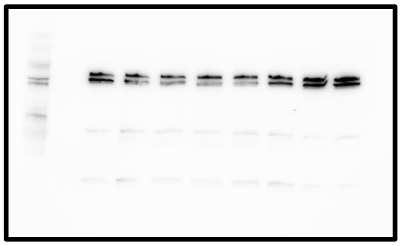

β-tubulin

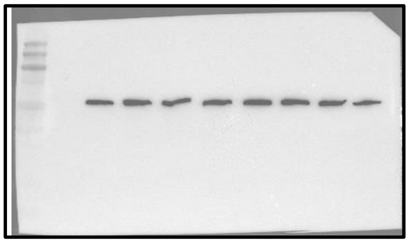

Figure 3B

CD73

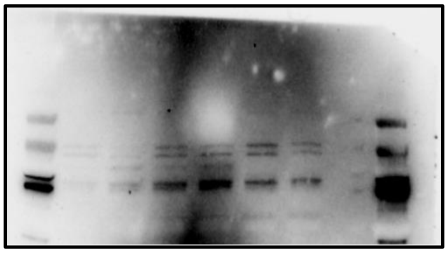

β-tubulin

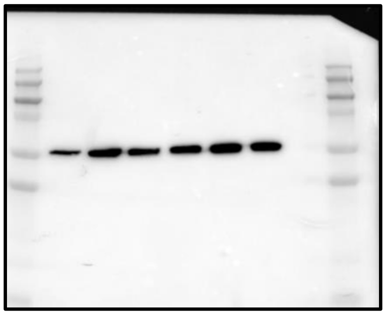

Figure 4A

OXPHOS complexes

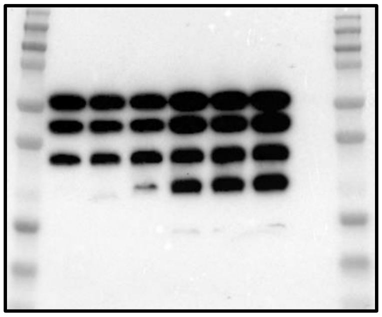

Histone 3

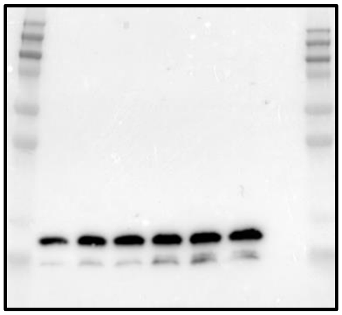

Figure 3E

ATP6V0D2

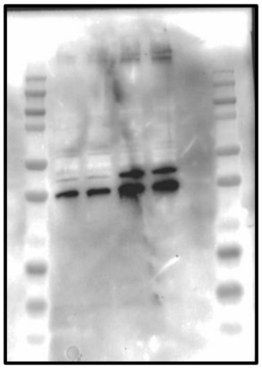

β-tubulin

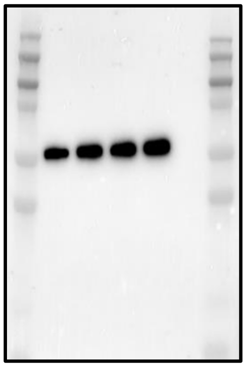

Figure 3E

Cathepsin K

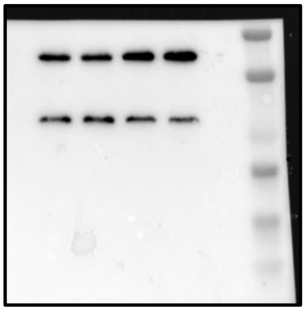

GAPDH

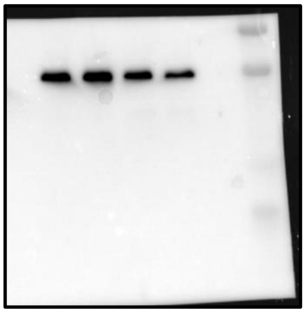

Figure 4H

Catalase

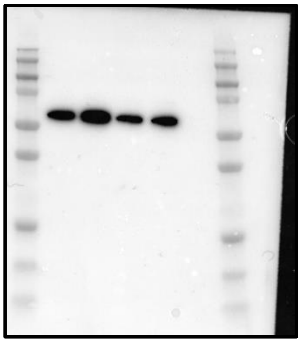

β-actin

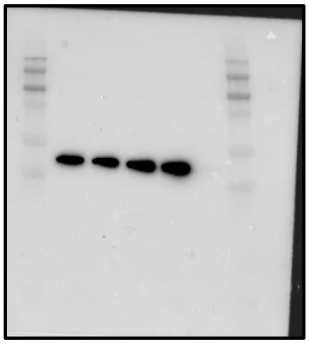

Supplementary Figure 1. Original uncropped western blots used for preparation of Figure 1P (MMP-13 and β-tubulin), Figure 3B (CD73 and β-tubulin), Figure 3E (ATP6V0D2, β-tubulin, Cathepsin K, GAPDH), Figure 4A (OXPHOS protein complexes and Histone 3), Figure 4H (Catalase and β-actin).
